# Generating learning guides for medical education with LLMs and statistical analysis of test results

**DOI:** 10.1186/s12909-025-06978-2

**Published:** 2025-03-29

**Authors:** Iván Roselló Atanet, Mihaela Tomova, Miriam Sieg, Victoria Sehy, Patrick Mäder, Maren März

**Affiliations:** 1 Charité - Universitätsmedizin Berlin, corporate member of Freie Universität Berlin and Humboldt Universität zu Berlin, AG Progress Test Medizin, Charitéplatz 1, 10117 Berlin, Germany; 2 Fakultät für Informatik und Automatisierung, Data-Intensive Systems and Visualization Group (dAI.SY), Technische Universität Ilmenau, Ehrenbergstraße 29, 98693 Ilmenau, Thuringia Germany; 3 Fakultät für Biowissenschaften, Friedrich Schiller Universität Jena, Schloßgasse 10, 07743 Jena, Thuringia Germany

**Keywords:** Natural Language Processing, Large Language Models, MeSH terms, Feedback, Education

## Abstract

**Background:**

The Progress Test Medizin (PTM) is a formative test for medical students issued twice a year by the Charité-Universitätsmedizin Berlin. The PTM provides a numerical feedback based on a global view of the strengths and weaknesses of students. This feedback can benefit from more fine-grained information, pinpointing the topics where students need to improve, as well as advice on what they should learn in light of their results. The scale of the PTM, taken by more than 10,000 participants every academic semester, makes it necessary to automate this task.

**Methods:**

We have developed a seven-step approach based on large language models and statistical analysis to fulfil the purpose of this study. Firstly, a large language model (ChatGPT 4.0) identified keywords in the form of MeSH terms from all 200 questions of one PTM run. These keywords were checked against the list of medical terms included in the Medical Subject Headings (MeSH) thesaurus published by the National Library of Medicine (NLM). Meanwhile, answer patterns of PTM questions were also analysed to find empirical relationships between questions. With this information, we obtained series of questions related to specific MeSH terms and used them to develop a framework that allowed us to assess the performance of PTM participants and compose personalized feedback structured around a curated list of medical topics.

**Results:**

We used data from a past PTM to simulate the generation of personalized feedback for 1,401 test participants, thereby producing specific information about their knowledge regarding a number of topics ranging from 34 to 243. Substantial knowledge gaps were found in 14.67% to 21.76% of rated learning topics, depending on the benchmarking set considered.

**Conclusion:**

We designed and tested a method to generate student feedback covering up to 243 medical topics defined by MeSH terms. The feedback generated with data from students in later stages of their studies was more detailed, as they tend to face more questions matching their knowledge level.

## Introduction

The Progress Test Medizin (PTM) is a formative test for medical students assembled twice a year since 1999 by the Charité-Universitätsmedizin Berlin; as of 2024, it is administered in two languages (German and French) by 17 universities in Germany, Austria and Switzerland. The PTM consists of 200 multiple-choice questions at graduate level. The content of each PTM question refers to one or more domains, organ systems and subjects and can be either of theoretical or practical nature.

The main goal of the PTM is to help students measure their knowledge across all semesters and curricula, providing them with a cross-sectional and longitudinal assessment. Some faculties in the PTM consortium ask test takers to indicate the level of certainty of their responses through a Likert scale with three items (“sure”, “likely” and “guessed”).

Currently, PTM test takers receive a six-page report shortly after the test, containing information about their test outcomes, how they compare to peers in the same cohort and faculty, a breakdown of results by subject and organ system, and data on how their scores have progressed over time.

However, feedback documents provided by the PTM consortium do not include yet any learning advice on how to improve. It is therefore convenient to enhance the currently provided numerical feedback with more personalized information based on PTM question content and test results.

Building on Sehy et al.’s work on the feedback preferences of PTM participants [1], we further investigated feedback content, structure, and use. The literature shows that students prefer specific feedback over simple right-wrong answers [1–3], a clear indication of strengths and weaknesses [1], and a stronger link to questions relevant to the German *Staatsexamen* in the case of the PTM [1].

Joseph et al. [4] showed that structured, formative feedback combined with concept maps improves learning, as students revised maps based on feedback and extra materials. In addition, students want to use feedback for revision [2], self-improvement [1], and comparing their performance with peers [1].

In general, preparing feedback is a time-consuming task, with its quality often depending on the person preparing it and the size of the test for which the feedback is being created. Additionally, when multiple individuals are involved in preparing feedback, discrepancies may arise depending on what each person considers important to include. Furthermore, preparing personalized feedback involves collecting and interpreting detailed performance data, considering the strengths, weaknesses, learning styles, and preferences of students within the framework of a consistent feedback procedure.

We thus aim to offer a way to provide students with feedback that follows a specific structure, is personalized to their performance, focuses on identifying possible knowledge gaps, and offers learning advice on what to study and in what order of priority, while also taking into account the preferences expressed by students in this regard.

Under the assumption that learning in medical education is usually incremental and cumulative, i.e., students are expected to learn new concepts based on what they have already learned, our feedback procedure is developed from the definition of concept maps as graphs that model the progression of students across the sequences of learning topics determined by their curricula. We have expanded this idea into a methodological structure based on three main elements: large language models, the MeSH thesaurus, and the statistical analysis of PTM results.

Large language models such as ChatGPT are deep neural network models trained on vast data, including books, articles, and websites; they are used for tasks like text generation, translation, summarization, and question answering, making them especially interesting for the education field.

In order to be able to provide effective feedback targeting gaps in the knowledge of students, it is essential to first have an overview of how PTM questions relate to each other. To achieve this, we must find and link similar content between PTM questions and associate it to a controlled vocabulary that ensures consistency in the terminology used to define learning topics. In our work, we used the MeSH thesaurus for that purpose.

The MeSH thesaurus is a controlled, hierarchical vocabulary produced by the United States National Library of Medicine (NLM) and used for indexing, cataloguing, and searching of biomedical and health-related information [5]. The MeSH thesaurus contains three types of records: descriptors, qualifiers and supplementary concept records [6, 7]. MeSH descriptors are organized hierarchically into up to 13 levels; their position in this hierarchy is indicated by a tree number. MeSH descriptors can occupy more than one position in the hierarchy; therefore, some MeSH terms are assigned multiple tree numbers [8]. This results in a rooted tree graph where edges represent the relation “is a subset of”, and nodes correspond to tree numbers. This structure allows the MeSH thesaurus to benefit from the mathematical properties of rooted trees.

The use of MeSH terms in the educational domain is not uncommon. In the work of Majernik et al. [9] the authors implement the online platform EDUportfolio and use MeSH terms as part of its evaluation. In the work of Hege et al. [10], the authors have designed a clinical reasoning tool that lets students visualize virtual patient’s illnesses as a concept map that can be compared to experts’ reasoning via the use of MeSH terms.

Finally, the statistical analysis of PTM results provides real data about how students actually deal with the topics they are supposed to learn. The clustering procedure described in [11] allows us to structure this information so that the recommendations that PTM participants receive as part of their feedback are appropriate to their knowledge levels.

Given the importance of preparing qualitative feedback and the field for which we want to prepare it, we define the following research questions to guide our work:

1. *How can artificial intelligence be leveraged in the design of personalized feedback from PTM test content?* We answer this research question by first using the LLM ChatGPT 4.0 to extract useful information from PTM questions in the form of medical terminology (MeSH terms) in order to link them based on relevant content overlap between them.
2. *How can we assess output generated by LLMs based on PTM content?* We assess the output produced by ChatGPT 4.0 against the MeSH thesaurus. We expand this output further by incorporating the hierarchical structure of this thesaurus.
3. *How can PTM empirical results and content similarity between questions be used to construct learning guides?* We divide PTM items into question groups that follow a framework consisting of “topical” and “empirical” relationships. Topical relationships are based on the occurrence of common terms among the sets of MeSH Terms associated to different questions, while empirical relationships are determined according to each item´s response patterns. To this end, we introduce the notion of *precursor question*, which allows us to identify pairs of empirically related test items.

Our study is the first to propose an approach that provides students with personalized feedback in the form of learning guides targeting content from the PTM.

## Methods

### Approach

We propose in this manuscript a seven-step approach to automate the construction of personalized feedback. Our approach is based on the use of a large language model to match the content of PTM questions to a controlled vocabulary, followed by statistical analysis aimed at finding empirical relationships between PTM questions, from which the guided learning advice will be generated. We chose to use ChatGPT 4.0, developed by OpenAI and available at the time of writing as a subscription-based service on OpenAI’s website [12]. Any other LLM with similar performance can be used instead.

We used Python (3.9.18) and the Python library scikit-learn (1.4.1) for performing data analysis, and Plotly (5.13.0) for generating examples of visual feedback.

**Fig. 1:**
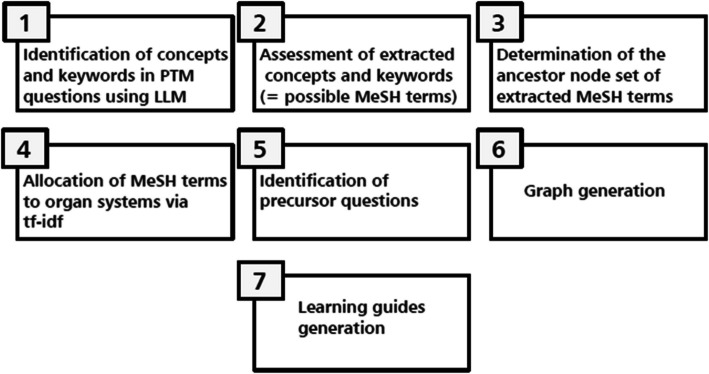
Overview of the proposed approach

An overview of this approach is shown in Fig. 1.

### Dataset

For this study, we have used data from the 46th issue of the PTM (PT46) conducted at the Charité-Universitätsmedizin Berlin. Our dataset contains 2,932 participations, corresponding to test takers who answered at least one question; students in their “practical year” (junior residency) were excluded.

### Step 1: identification of underlying concepts and keywords in PTM questions using a large language model

**Fig. 2:**
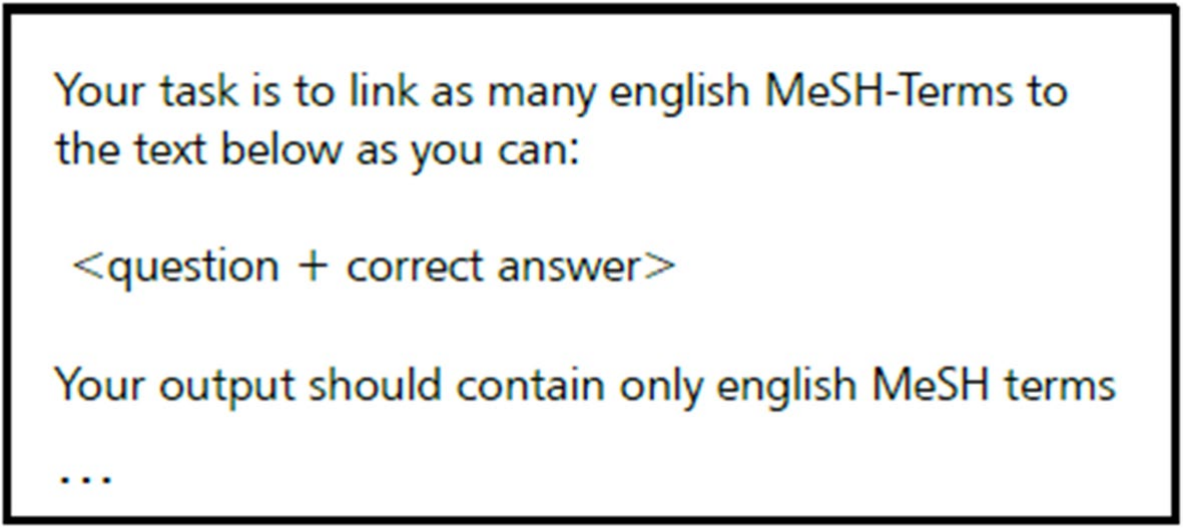
ChatGPT prompt used to extract MeSH terms

We prompted ChatGPT 4.0 to identify concepts and keywords in the form of English MeSH terms from PTM questions in the German language. The prompt we used is presented in Fig. 2.

Our prompt followed the tactic “Include details in your query to get more relevant answers” as proposed in Open AI’s guides [13]. In our prompt, we specified that the LLM should generate MeSH terms in English based on a given PTM question in German. For every specific question, we gave its question vignette and correct answer as textual input; distractors were excluded, as their inclusion could lead to establishing wrong links between questions. For example, let (A,B) be a pair of questions such that “diabetes mellitus” is the correct answer for A and a distractor for B. Then, if distractors were considered, the algorithm could link these two questions and use them to evaluate diabetes-related knowledge even if "diabetes mellitus" is not that relevant to question B. By including only the question vignette and the correct answer, we can be hundred per cent sure that we are working with relevant content.

**Fig. 3:**
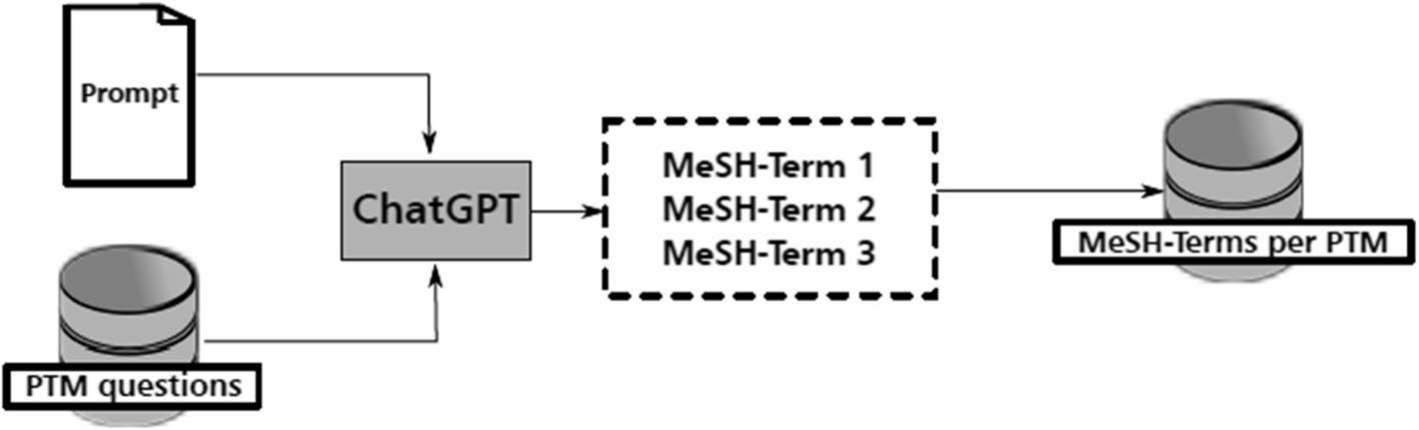
Overview of step one: ChatGPT extracts MeSH terms from PTM questions, which are then stored in a database

We summarize the process in Fig. 3.

### Step 2: assessment of the extracted MeSH terms

Due to the probabilistic nature of LLMs, we prompted ChatGPT 4.0 to extract MeSH terms three times for the same PTM question to maximize the number of terms and observe the variations in each output.

To evaluate the differences among the three outputs, we first reduced all keywords to their base or dictionary form – a procedure known as lemmatization—and calculated cosine similarity scores between the outputs. We then combined the extracted concepts and keywords from all three outputs, removing duplicates. Finally, we verified the outputs by matching them to MeSH terms, i.e., descriptors and entry terms in the NLM’s MeSH thesaurus, which required the LLM to output the terms in English.

**Fig. 4:**
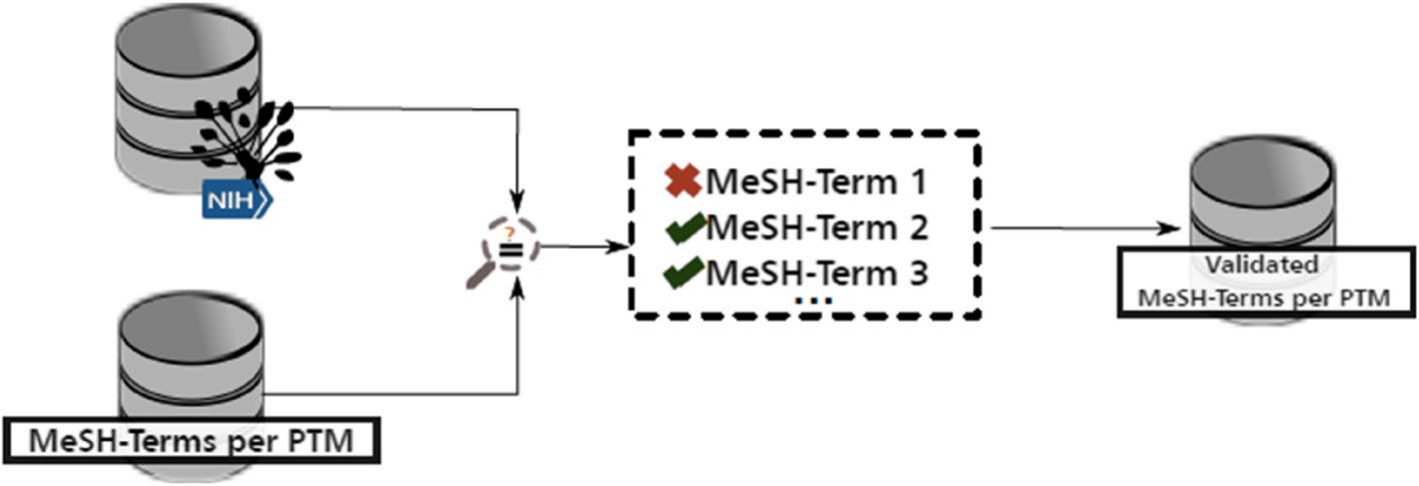
Overview of step two: The extracted MeSH terms are compared to those from the NLM´s MeSH thesaurus, and the validated terms are stored in a database

We summarize this step in Fig. 4. More information about this procedure is provided in Appendix B.

### Step 3: determination of the ancestor node set of every extracted MeSH term

Since each MeSH term defines a subset of their ancestor terms, one could assume that a question associated to a given MeSH term is also associated to its ancestors. Thus, we enhanced every set ${s}_{i}\in S$ of MeSH terms associated to a question *q* with a set ${A}_{i}$ of ancestors ${a}_{i,1},\dots ,{a}_{i,j\left(i\right)}$ where $j(i)$ is the total number of ancestors of the terms contained in the set ${s}_{i}$. Hence ${s}_{i}={s}_{i}U{A}_{i}$.

**Fig. 5:**
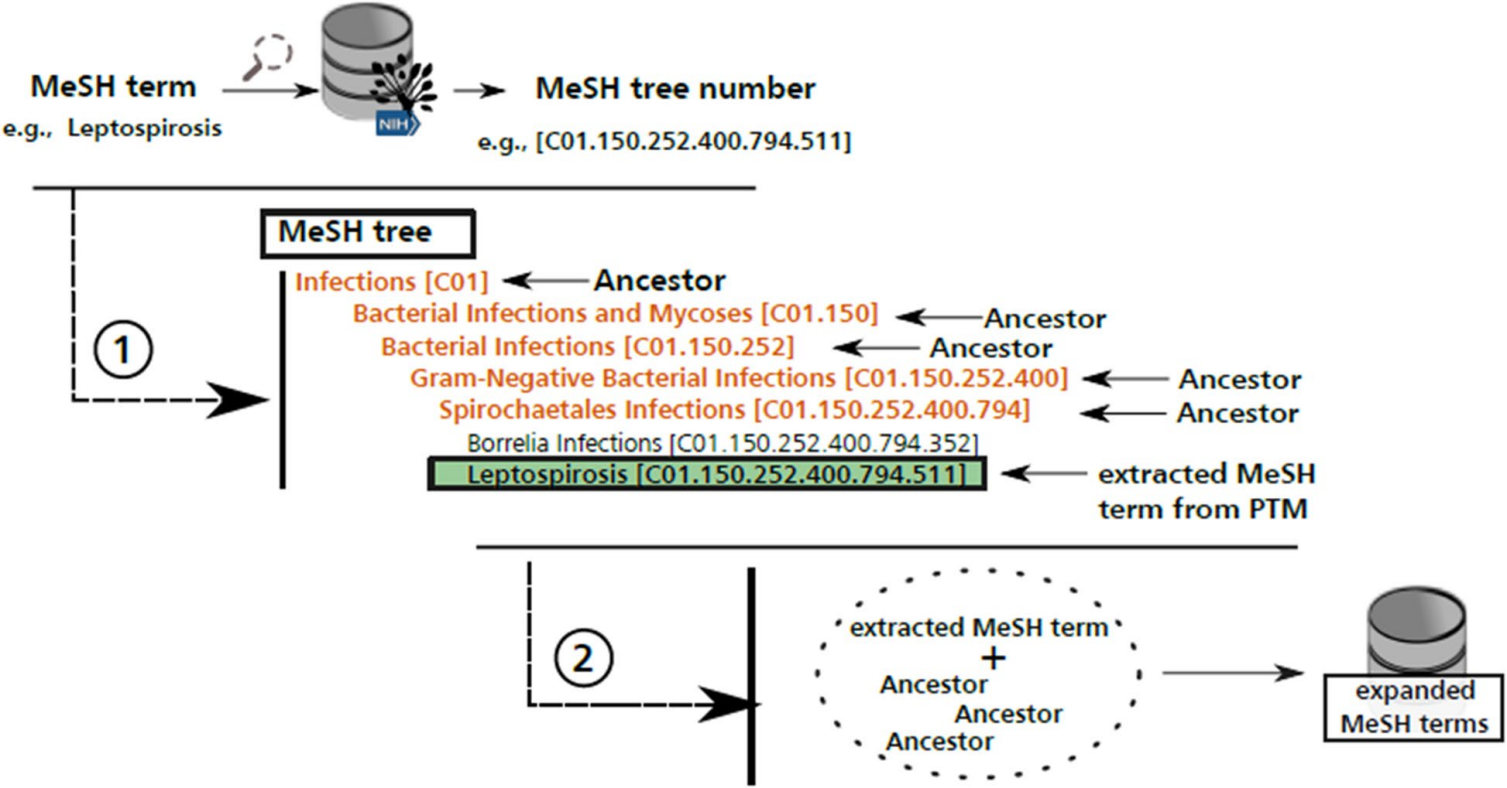
Overview of step three: Validated MeSH terms are extended with ancestor MeSH terms identified in the MeSH thesaurus

We summarize this step in Fig. 5.

### Step 4: allocation of MeSH terms to organ systems using tf-idf

Our method is meant to offer 14 learning guides per participant, one for each organ system included in the PTM blueprint [14].

After identifying MeSH terms from PTM questions, we wanted to find the most relevant ones for each organ system [14].

We used tf-idf [15], a statistic used in information retrieval to measure the relative importance of words to documents, to gauge how relevant MeSH terms are to organ systems, treating organ systems as documents and MeSH terms as words. For any given organ system, the MeSH terms selected were the ones whose tf-idf value was higher than the mean for that organ system plus one standard deviation. More information about this procedure can be found in Appendix C.

**Fig. 6:**
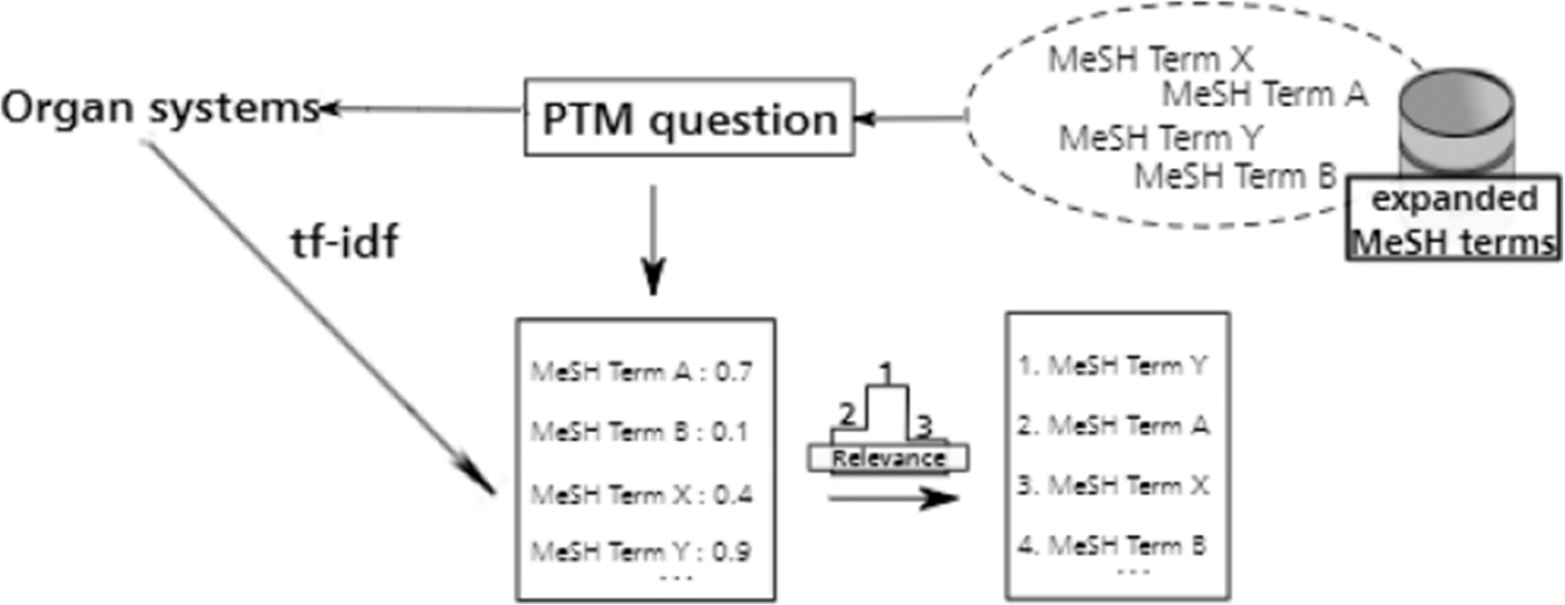
Overview of step four: For each PTM question, relevance scores are computed via tf-idf between MeSH terms and organ systems associated with the questions

We summarize this step in Fig. 6.

### Step 5: identifying precursor questions

We intend to capture the relationships between sets of PTM test questions and answers and use this information to offer students useful feedback in the form of learning guides. As a complement to the content-based similarities between questions that we have explored up to this point, we decided to examine the similarities between answer patterns of different PTM questions.

In particular, we found it interesting to explore to what extent knowing the answer to a given question *A* might be a precondition to knowing the answer of a different question *B*. We formalise this relationship by introducing the concept of *precursor question*, which is essentially an adaptation of the relative risk index to the context of a multiple-choice test; Cohen’s *d* is then used to measure the strength of this relationship between questions. On a conceptual level, precursor questions can also be seen as a concrete implementation of the notion of surmise question posited by Doignon and Falmagne [16], albeit based primarily on operational needs rather than on theoretical considerations.

**Fig. 7:**
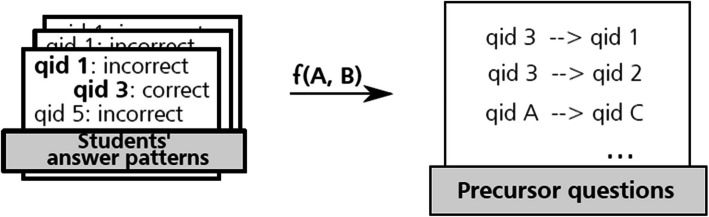
Overview of step five: relations between PTM questions are established based on answer patterns

The fundamentals of precursor questions are explained in detail in Appendix A. We summarize this step in Fig. 7.

### Step 6: graph generation

#### General framework

Our goal is to generate individual learning guides for each of the 14 organ systems in the PTM catalogue. These learning guides will be generated from a graph containing suitable pairs of questions (*A,B*), both of them associated with the organ system for which the learning guide is generated.

#### Path generation

Each MeSH term *t* allocated to an organ system with tf-idf generates an initial path ${p}_{initial}={\{q}_{1},..,{q}_{m}\}$ containing the *m* questions belonging to the organ system *s* whose content is related to the MeSH term *t*. Questions ${q}_{1},..,{q}_{m}$ were ordered from easiest to most difficult**;** question difficulty was determined by computing the share of correct answers among all responses provided by test participants.

From each initial path ${p}_{initial}={\{q}_{1},..,{q}_{m}\}$ we generated a path $p={\{q}_{1},..,{q}_{n}\} , n\le m$, containing the longest question sequence $S={\{q}_{1},..,{q}_{n}\}$ such that ${q}_{i}$ is a precursor question of ${q}_{i+1}$ for all $i$ such that$1\le i<n$*.* If there were two or more sequences ${S}_{1},..,{S}_{j}$ with maximum length, we chose among them the sequence ${S}_{i}$ such that $max(f({q}_{i}, {q}_{i+1}), 1\le i< n,{q}_{i}\in {S}_{i}) =min(max(f({q}_{i}, {q}_{i+1}), 1 \le i<n, {q}_{i} \in {S}_{k}, 1\le k<j )$, that is, the sequence with the lowest maximum value of the precursor question function among its consecutive elements. Hence all paths $p={\{q}_{1},..,{q}_{n}\}$ thus generated are sequences of precursor questions defined by some MeSH term.

**Fig. 8:**
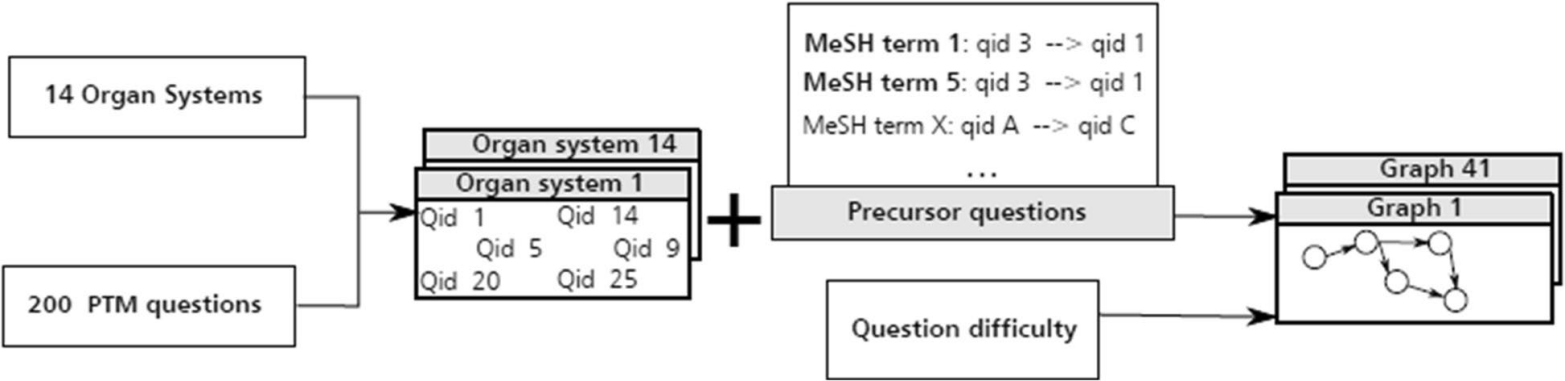
Overview of step six: Graphs are constructed on the basis of organ systems, precursor questions, and question difficulty

We summarize this step in Fig. 8.

### Step 7: generation of learning guides

#### Generating the visual representation

Given that the PTM is administered to students across different semesters and knowledge levels, and considering its longitudinal nature, we want that our visual feedback enables students to compare their performance with peers at the same level and identify what they need to learn to progress to the next stage.

To this end, we used the clustering procedure described by Sieg et al. [11]. PTM test takers were grouped into five clusters based on response patterns, confidence levels, and total scores. Three “performance” clusters (cluster 0, cluster 1, and cluster 3) and two “drop-out” clusters (cluster 2 and cluster 4) were then identified. As stated in the study of Sieg et al., students in cluster 2 typically showed above average performance in the first half of the test but did not complete the second half, while cluster 4 included mostly first-year students and “non-serious” participants who provided very few answers or guessed most of the test. On the other hand, participants in “performance” clusters usually tended to complete the test; this is reflected in the median number of omitted items per cluster, which in the study of Sieg et al. amounted to 0 for all three “performance” clusters, but 107 (53.5%) for cluster 2 and 126 (63%) for cluster 4. This means that we have to assume that most participants included in the “drop-out” clusters failed to provide reliable information about their knowledge level via the PTM.

Let ${r}_{t}$ be the mean share of correct answers of the test *t*, and let *Q* be a question of the test *t* with shares of correct answers ${r}_{Q,0},{r}_{Q,1},\dots ,{r}_{Q,i}$ for a set of clusters 0,1,…,*i*, ordered from best to worst performance. Let ${C}_{Q}=\{j\in \{0,..,i\},{r}_{Q,j}>{r}_{t}\}$ be the subset of clusters for which the share of correct answers to *Q* is higher than *r*_*t*._ Then *Q* will be assigned to ${max(C}_{Q})$*,* that is, to the worst performing cluster where the mean share of correct answers exceeds ${r}_{t}$. This is intended to represent the minimum knowledge level required to successfully answer question *Q*. Questions such that the set ${C}_{Q}$ is empty—that is, that their share of correct answers is lower than ${r}_{t}$ for all clusters—will not be assigned to any cluster.

With this in mind, our visual feedback will first classify students into “performance” clusters, indicating their current knowledge level. As described in steps 3 to 6 of our approach, we will generate graphs based on precursor questions, question difficulty scores, and organ systems. Each node will represent a PTM question and will be colour-coded to reflect the knowledge level (cluster) typically required to answer it correctly. Our visual feedback will also show whether a student answered a question correctly or not and their level of confidence in each answer, helping them identify areas that need more attention; if a student answers a question correctly at their knowledge level but lacks confidence, this may indicate a knowledge gap. Colour coding will also help students quickly focus on questions and topics that are relevant for their knowledge level before moving on to more advanced material.

**Fig. 9:**
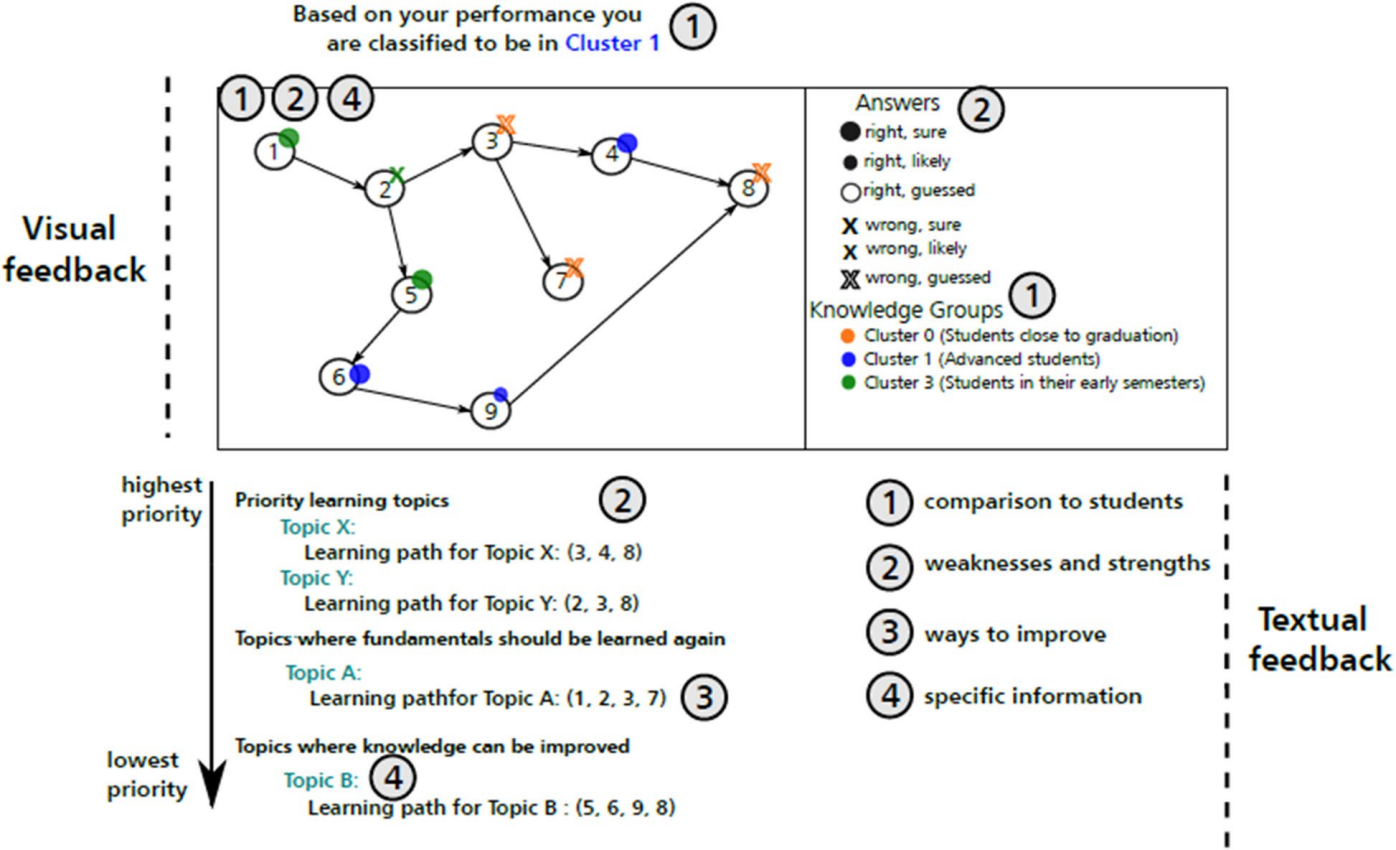
Overview of learning guide feedback

An overview of the visual feedback is shown in Fig. 9.

#### Generating the textual part

According to the literature, high-quality feedback should highlight strengths and weaknesses, offer guidance for improvement, and provide detailed information on the relevant content. Our textual feedback is intended to meet these requirements.

The MeSH term-based topics provide students with detailed insights into the content covered in the PTM, and by prioritizing them, each student’s strengths and weaknesses will be highlighted. Each linked question may connect to additional topics, providing students with opportunities to broaden their knowledge, particularly in areas where they struggled to answer correctly.

We prioritize the learning topics for each student by applying the following strategy: Let *t* be a mesh term, $${S}_{t}={\{q}_{1},..,{q}_{n}\}$$ its associated question sequence for a given organ system, and *p* an individual test participant belonging to cluster $${c}_{p}$$. Every question $${q}_{i}$$ in $${S}_{t}$$ is also allocated a cluster $${c}_{q,i}$$ depending on its difficulty level (see above). Then we can divide questions into three groups according to their difficulty level relative to the knowledge of participant *p*: basic questions ($${c}_{q,i}>{c}_{p})$$, current level questions ($${c}_{q,i}={c}_{p})$$ and higher level questions ($${c}_{q,i}<{c}_{p})$$,.

Both basic and current level questions are supposed to fall into the scope of what participant *p* may know. Yet there is a difference between these two levels. On one hand, current level questions should correspond to what participant *p* is learning at the moment, implying that the process of acquiring these contents might be incomplete. Therefore, participant *p* is not necessarily expected to know the answers to all questions at this level. On the other hand, basic questions are related to a previous degree stage that participant *p* has theoretically seen through, so a failure to respond these questions correctly might reveal forgotten or misunderstood basic concepts.

Higher level questions are supposed to be beyond the current knowledge level of participant *p*. However, they could be useful to support recommendations to study some topics deeper, e.g., those where basic and current level questions were all answered correctly, but higher level questions were not.

For this analysis we considered only the MeSH terms whose related question sequences contained at least two basic or current level questions. According to these rules, we defined the following groups of learning topics:

- *Priority learning topics*: Topics for which less than 50% of the basic and current level questions were answered correctly.
- *Topics whose fundamentals should be learned again*: Topics for which at least one basic question was not answered correctly (excluding those classified in the “priority learning topics” group).
- *Topics whose knowledge can be improved*: Topics for which at least one current level question was not answered correctly (excluding those classified in the above groups).
- *Topics suitable for deeper study*: Topics for which all basic and current level questions were answered correctly, but at least one higher level question was not.
- *Topics without apparent knowledge deficits*: Topics for which all questions were answered correctly.

All learning guides generated in the context of this study are in English. However, these learning guides are planned to be multilingual, since PTM feedback is currently provided in both German (for faculties in Germany, Austria and German-speaking Switzerland) and French (for faculties in French-speaking Switzerland). More languages could be added in the future on request of participating faculties.

## Results

### Identification and assessment of extracted MeSH terms from PTM questions

We applied steps 1 and 2 of our approach on data from the 46th issue of the PTM (PT46). To account for the probabilistic nature of LLMs, we ran our model across three separate sessions. Our analysis shows that the number of concepts and keywords extracted by ChatGPT in a single session ranged most frequently between 1 and 5. The number of concepts and keywords found by question across all three sessions ranged from 2 to 23; the most frequent ranges were 6 to 10, 1 to 5, and 11 to 15, with modes of 6, 4, and 11 respectively.

To find out how similar the outputs of the three sessions were, we computed the cosine similarity between them. Their pairwise average similarity scores ranged from 82.8% to 84.4%.

The frequency distribution of concepts and keywords of the combined output is shown in Table 1; the average cosine similarity scores are presented in Table 2.

**Table 1:** MeSH terms split into ranges based on the number of MeSH terms generated by ChatGPT 4.0. Count shows the number of occurrences in each range, mode shows the most frequent number of MeSH terms in a range, and min and max show the smallest and the largest number of MeSH terms in a range

**Session 1**				
MeSH terms range	count	mode	min	max
**1—5**	100	4	2	5
**6—10**	89	6	6	10
**11—15**	10	11	11	15
**16—20**	1	20	20	20
**21—23**	-	-	-	-
**Session 2**				
MeSH terms range	count	mode	min	max
**1—5**	101	5	2	5
**6—10**	91	6, 7	6	10
**11—15**	7	12	11	15
**16—20**	1	19	19	19
**21—23**	-	-	-	-
**Session 3**				
MeSH terms range	count	mode	min	max
**1—5**	118	4	2	5
**6—10**	76	6	6	10
**11—15**	4	11,12,13,14	11	14
**16—20**	2	16,19	16	19
**21—23**	-	-	-	-
**Combined**				
MeSH terms range	count	mode	min	max
**1—5**	54	4	2	5
**6—10**	96	6	6	10
**11—15**	43	11	11	15
**16—20**	8	17,19	17	19
**21—23**	2	22,23	22	23

**Table 2:** Average cosine similarity between extracted concepts and keywords, computed between sessions

	Repeat 1 & Repeat 2	Repeat 1 & Repeat 3	Repeat 2 & Repeat 3	All
cos. sim. (average)	0.844	0.838	0.828	0.837
cos. sim. (mode)	0.863	0.867	0.849	

In order to be certain that the concepts and keywords produced by ChatGPT were actually related to their associated test items, we checked them manually for 28 randomly chosen questions (2 questions for each organ system), obtaining the following results: Out of 216 concepts and keywords found by ChatGPT for these 28 questions, 194 (89.81%) were directly featured or alluded in a question vignette or an answer, and a further 21 (9.72%) correspond to wider topics related to a question (for example, “thoracic surgery” for a question about chest tubes). Therefore, 215 out of 216 concepts and keywords (99.54%) were found to be related to the medical content of their associated questions; the remaining keyword is also related to its associated question, but refers to the scenario described in the vignette rather than to the medical topics actually covered in it.

Based on the combined outputs for all 200 questions, ChatGPT 4.0 identified 1,639 occurrences of concepts and keywords, which implies a mean of 8.195 occurrences per question; the standard deviation was 3.926.

To find out whether the extracted concepts and keywords were indeed MeSH terms, we matched them against descriptors or entry terms found in the MeSH thesaurus. Out of the 1,639 concepts and keywords extracted, 169 (10%) did not match any descriptors or entry terms, while 1,354 (83%) fully matched descriptors included in the current MeSH thesaurus. A further 52 (3%) partially matched descriptors included in the current MeSH thesaurus, and another 20 (1%) fully matched entry terms. Finally, 44 (3%) partially matched entry terms. From this point on, we used only concepts and keywords that matched descriptors or entry terms included in the current MeSH thesaurus; the 169 concepts and keywords that did not match any descriptors or entry terms were discarded.

### Generation of learning guides

#### Distribution of test participations and questions according to clusters

As in the work of Sieg et al. [11], clusters 2 and 4 are also “drop-out” clusters in our analysis. Table 3 shows that the mean number of omitted responses was 6.28, 6.39 and 7.28 for clusters 0, 1 and 3, versus 93.1 and 114.41 for clusters 2 and 4. We conclude from this that it only makes sense to generate learning guides for test takers in clusters 0, 1 and 3, since the large number of omitted responses in clusters 2 and 4 indicates gaps in the data that the test is supposed to provide.

**Table 3:** Mean number of responses by cluster and type of response according to the Likert scale shown to participants in the PTM test

Cluster	Response						
	Sure, correct	Likely, correct	Guessed, correct	Sure, incorrect	Likely, incorrect	Guessed, incorrect	Not answered
**0**	85.09	39.76	19.7	10.53	16.6	22.04	6.28
**1**	48.23	43.56	29.23	9.1	24.27	39.22	6.39
**2**	25.71	20.79	17.91	4.56	12.29	25.63	93.1
**3**	25.05	27.99	33.08	13.25	28.15	65.19	7.28
**4**	5.36	6.53	15.61	5.78	11.03	41.27	114.41

The breakdown of participations by cluster and semester of study is shown in Table 4. If we do not consider participations allocated to clusters 2 and 4, cluster 3 predominates before the fifth semester, then cluster 1 is in the majority for semesters 5 to 7 and cluster 0 is dominant from the eighth semester onwards. Clusters 0, 1 and 3 might then be associated respectively with the advanced, intermediate and basic stages of a medical degree.

**Table 4:** Distribution of participations in the dataset according to their semester of study and the cluster they were assigned

Cluster	Semester										
	**1**	**2**	**3**	**4**	**5**	**6**	**7**	**8**	**9**	**10**	**Total**
**0**	0	0	2	7	20	37	29	56	85	87	**323**
**1**	4	14	26	57	96	80	75	49	59	39	**499**
**2**	2	3	19	27	56	45	54	59	52	43	**360**
**3**	114	129	136	114	48	15	12	3	5	3	**579**
**4**	194	182	147	116	107	110	109	101	52	53	**1171**
**Total**	**314**	**328**	**330**	**321**	**327**	**287**	**279**	**268**	**253**	**225**	**2932**

Regarding the breakdown of questions by allocated cluster, 55 questions were assigned to cluster 3; 63 questions were assigned to cluster 1, and 54 questions were assigned to cluster 0. Finally, there are 28 questions whose difficulty level places them far beyond the usual knowledge of PTM test takers; they were not assigned to any cluster. This means that the number of approachable questions amounts to 55 for participants in cluster 3 (27.5%), 118 for participants in cluster 1 (59%) and 172 for participants in cluster 0 (86%). The comparatively scarce number of questions that students in cluster 3 can successfully answer is thus reflected in the number of topics rated per cluster, which amounts to 243 for cluster 0, 154 for cluster 1 and only 34 for cluster 3.

#### Distribution of topics according to student clusters and type of recommendation

Among the topics actually rated, we see that the sum of the groups “Topics without apparent knowledge deficits” and “Topics suitable for deeper study” accounts for 35% to 45% of all topics for the three clusters analysed (42.62% for cluster 0, 37.22% for cluster 1, and 37.74% for cluster 3). These two groups include the topics for which students have answered all the questions associated to both their cluster and its lower-level clusters. Therefore, their knowledge about these topics is in line with expectations. Indeed, the group “Topics without apparent knowledge deficits” includes topics for which students have answered all questions correctly regardless of their difficulty. Unsurprisingly, students in cluster 0 show a clear lead here over those in clusters 1 and 3. The groups “Topics whose knowledge can be improved” and “Topics whose fundamentals should be learned again”, which stand for non-severe knowledge gaps, account for 40% to 45% of all topics (42.62% for cluster 0, 42.52% for cluster 1 and 40.53% for cluster 3). Here we see some contrast between cluster 0 on one hand and cluster 1 on the other, because students in cluster 0 show a higher share of topics classified under “Topics whose fundamentals should be learned again”, which could mean that their knowledge gaps are more frequently associated with content they have learned earlier in their degree and might have forgotten; at the same time, students in cluster 1 tend to experience more difficulties with topics they are currently learning. Finally, the size of the group “Priority learning topics”, including the topics for which the most severe knowledge gaps were found, is smaller in cluster 0 (14.67% of all rated topics) and larger in cluster 1 (20.26%) and cluster 3 (21.76%).

#### Most frequent priority learning topics

We have identified the most frequent priority learning topics for every cluster; as we can see in Table 5, for cluster 0 these were Musculoskeletal and Neural Physiological Phenomena and Muscles, both of which were recommended as priority learning topics to 149 test takers, or 46.13% of all test takers in cluster 0. The most frequent priority learning topic for cluster 1 was Circulatory and Respiratory Physiological Phenomena, recommended as priority learning topic to 279 test takers, or 55.91% of all test takers in cluster 1. For cluster 3, the most recommended priority learning topic was Health Occupations, suggested to 297 test takers, or 51.3% of all test takers in cluster 3.

**Table 5:** Learning topics most frequently classified as “priority learning topic” in the organ system-based feedback, by student cluster. We have listed the 10 most recommended topics for each cluster; the list for cluster 3 includes 12 topics, since there were five of them tied at eighth place

Cluster 0			Cluster 1			Cluster 3		
**MeSH Term**	**Organ system**	**Count**	**MeSH Term**	**Organ system**	**Count**	**MeSH Term**	**Organ system**	**Count**
Musculoskeletal and Neural Physiological Phenomena	Musculoskeletal System	149	Circulatory and Respiratory Physiological Phenomena	Cardiovascular System	273	Health Occupations	Methodology, Instruments	297
Muscles	Musculoskeletal System	149	Aged	Cardiovascular System	269	Digestive System Diseases	Digestive System	250
Autoimmune Diseases of the Nervous System	Nervous System, Brain, Senses	147	Arrhythmias, Cardiac	Cardiovascular System	269	Mental Disorders	Digestive System	224
Female Urogenital Diseases	General Pathology	143	Nutritional and Metabolic Diseases	Hormones, Metabolism	253	Behavioral Symptoms	Mind, Social Issues	193
Reproductive Physiological Phenomena	Reproductive Organs	143	Brain Diseases	Nervous System, Brain, Senses	248	Bacteria	General Pathology	188
Gastrointestinal Tract	Digestive System	140	Neurocognitive Disorders	Nervous System, Brain, Senses	248	Immune System Diseases	Blood, Lymph, Immune System	169
Myocardial Ischemia	Cardiovascular System	139	Musculoskeletal System	Musculoskeletal System	247	Immunoglobulin E	Blood, Lymph, Immune System	169
Guillain–Barre Syndrome	Nervous System, Brain, Senses	129	Tissues	Musculoskeletal System	247	Lung Diseases	Respiratory Organs	152
Peripheral Nerves	Nervous System, Brain, Senses	128	Ultrasonography	Reproductive Organs	240	Hemodynamics	Respiratory Organs	152
Neoplasms by Histologic Type	Cell	128	Pregnancy Complications	Reproductive Organs	235	Cardiovascular Physiological Phenomena	Respiratory Organs	152
						Heart Function Tests	Respiratory Organs	152
						Vital Signs	Respiratory Organs	152

## Discussion

### Answers to research questions

We formulated the following three research questions to guide our work:

- *Research question 1* investigates how artificial intelligence can be leveraged in the design of personalized feedback from PTM test content. Effective feedback should provide clear information about the knowledge that students are expected to possess, also highlighting possible knowledge gaps. In order to construct such feedback from PTM results, one needs to determine how the different concepts addressed in the questions relate to each other. In addition, students would like their feedback to be tailored to their knowledge level. Due to the scale of the PTM, these tasks must be automated; to assist educators in this endeavour we proposed using LLMs.
  Thanks to the capabilities of LLMs in various natural language processing tasks, as well as their knowledge in the medical field and other areas, we were able to semantically trace questions, allowing us to identify common concepts among them. This step was essential in our approach since it allowed us to identify faster, easier, and more precisely how questions relate to each other. Furthermore, by providing a more detailed overview of the topics in each question, we were able to pinpoint exact topics where students have possible deficits. Such additional information about questions can offer test-makers a different way to construct future tests, as they will have more topic-specific information about each question.
- *Research question 2* investigates how the content extracted by an LLM from a given multiple choice PTM question can be assessed. Given the field for which we want to construct feedback, it is important to assess the quality of the produced outputs by LLMs, since they are known to hallucinate information. Thus, we extracted concepts and keywords from PTM questions by using ChatGPT 4.0 and matched them to the NLM MeSH thesaurus. Our results indicated that 90% of the keywords found by ChatGPT were indeed MeSH terms that helped us find contextual links between PTM questions.
- *Research question 3 *asks how PTM empirical results and content similarity between questions can be used to construct learning guides.

Our feedback model is the result of considering two dimensions in relation to a given test and its questions, namely the topical dimension and the empirical dimension. The topical dimension refers to the subject matter covered by individual questions of the test and the extent to which this subject matter is similar between questions or not. It is the main element in previous examples of concept maps [17].

Since we are working with real test results, we can introduce a second axis that we call empirical dimension. This dimension refers to the process of knowledge acquisition by students, reflected on test results for individual questions. From this perspective, the empirical realization that knowing the answer to a given question significantly increases the probability of knowing the answer to another question might point to a relationship between their respective learning processes. This relationship may or may not be content based. When two questions are unrelated regarding their subject matter, any hypothetical closeness between their response patterns might be anecdotal or caused by factors beyond the scope of this study. We thus conclude that two questions that are empirically connected should also be topically connected in order to be relevant for the construction of learning guides.

Under these assumptions, we obtain learning guides whose recommended study topics are chosen according to the questions where they appear (part of the topical dimension) and the results of individual test takers in these questions are compared to those of a cluster of test takers with similar results. Furthermore, the questions associated to each topic were sequenced as series of precursor questions.

In the end, our approach was successful in identifying knowledge gaps and problem areas in some groups, such as participants from advanced semesters that showed deficits in topics taught in earlier semesters.

### Applicability of the method to other educational settings

We have conceived our method to be also applicable in other faculties or progress tests, assuming that some adaptations might be necessary due to possible differences regarding academic curriculum, internal organization, and composition of the student body among other factors. However, while we do not expect our methodology to be copied verbatim in case it is applied elsewhere, we would like to refer to some points worth paying attention when transferring our method to another context.

As stated in the title of this study, our method is based on two main elements: large language models on one hand, and statistical analysis of test results on the other. The first element brings together Chat GPT 4.0 and the MeSH thesaurus. Both are widely available; therefore, other educational institutions could use them as we have done. In any case, it is advisable that the MeSH terms yielded by the large language model are grouped according to a pre-existing framework; in this study, this role is played by the organ systems of the PTM, which we chose because they form the basis of the current undergraduate curriculum at the Charité- Universitätsmedizin Berlin. Medical disciplines can also be used to group MeSH terms together; the point is to have a structure that allows MeSH term-based feedback to be adapted to the curriculum of the faculty that implements the method.

The second element – statistical analysis of test results – must be treated more carefully, as it generates the necessary data to provide individualized feedback to students. Our statistical analysis of test results includes two subsequent procedures – precursor question analysis and clustering of tests. In our case, the precursor question analysis has benefited from the availability of a sizeable amount of test data, since the PTM includes 200 questions to which 2,932 test participants were asked to submit an answer. Even if not all of them did, this enabled us to construct a complex web of relationships between questions that might be somewhat different in the case of a smaller scale test.

In addition, our method relies on samples of questions and test takers that are sufficiently heterogeneous to highlight differences in learning. This is required to map the knowledge of students from the beginning to the end of their studies; if we only measure the performance of students having attained a certain level of knowledge (e.g. advanced students), the transition between different knowledge levels cannot be detected by statistical analysis. For example, the clustering of test participations will only yield relevant results if the response patterns of students are numerically separable into clearly differentiated clusters. For the same reason, the set of questions should contain items adapted to a wide range of knowledge levels; the idea of precursor questions as described in this study would not be meaningful for a sample of questions having similar difficulty.

In summary, we believe that adapting our method to other tests should be possible with the help of a structure that connects the MeSH terms to the academic curriculum of the organizing institution, as well as samples of questions and test participants that are sufficiently diverse and cover all stages of the academic programme to be assessed.

## Limitations

### Functionality of large language models

The usage of large language models in this study is restricted to natural language processing tasks such as finding keywords in PTM questions; on the other hand, our method generates student feedback automatically, but does so without using a large language model at that stage. This is because the automatic generation of feedback for the PTM implies grading the test first; a large language model would not be allowed to perform this task, since data protection regulations prevent feeding it with real test results. Moreover, Friederichs et al. [18] have found that the success rate of ChatGPT 3.5 in the task of choosing the correct answers to PTM questions is only 65.5%, which means that ChatGPT 3.5 cannot be trusted with grading the test. Even if we assume that ChatGPT 4.0 is more advanced than ChatGPT 3.5, we still cannot be sure that it would choose the correct answers to PTM questions with the required accuracy.

In addition, the automatic prompting of ChatGPT poses further difficulties that must be considered in the context of a test taken by 10,000 students each academic semester.

### Availability of the information required to construct the feedback

Our experiment in constructing personalized feedback in the form of learning guides showed that while we have enough information to produce actual MeSH term-based feedback for clusters 0, 1 and 3, this is not the case for cluster 2 and cluster 4. Cluster 2 encompasses students close to graduation who did not complete the whole PTM, while cluster 4 includes students who did not answer enough questions or are considered non-serious test-takers, who answer the questions randomly. In the case of clusters 2 and 4, we would not have enough history to prepare the feedback. For nonserious test-takers in cluster 4, feedback would be hard to construct and probably not very effective since these students pick answers and confidence levels at random. This might also happen to a lesser extent to students in cluster 3; since most questions of the PTM are yet beyond their knowledge, the data available to support the learning advice given by the model is scarce compared to clusters 0 and 1. As a direction for future research, we might suggest that this shortcoming could be mitigated by analysing whether failing certain types of questions in the early stages of the degree increases the likelihood of experiencing learning difficulties in later years.

A further limitation are the topic groups we formulated in our textual representation. These topic groups may vary depending on the knowledge of students and the field for which the feedback is prepared. In our case, we tailored the groups based on previous test data.

### Single-centre study

Although the PTM is conducted in 17 medical schools, we decided to work with data from only one faculty, as we wanted to track the performance of students following the same academic curriculum. It is technically possible to apply the method on data from other participating faculties, but we would have to consider them one by one because of possible curricular differences. In any case, we can apply our approach to test results from other participating faculties that provide similar data samples.

### Possible influence of poor questions

Inadequate multiple-choice questions might affect the quality of any knowledge assessment based on them, and the procedure described in this study is no exception to this rule. However, these questions can always be excluded from our test data provided that they can be identified with numerical procedures. For example, low-quality questions often deliver idiosyncratic answer patterns that bear little resemblance to those of other questions; based upon this, the precursor question part of the algorithm will filter them out. Furthermore, questions whose share of correct answers is too low will not be associated to any student cluster, so they will play a marginal role (if any) in the generation of learning guides. It is also possible to exclude questions with a lower discrimination index without compromising the performance of the algorithm.

## Conclusion

Constructing personalized feedback that motivates students and helps them improving their knowledge is not an easy task.

We propose a seven-step procedure that leverages a prominent LLM and statistical analysis to provide personalized learning advice for participants in the PTM, based on their responses to individual test questions.

We tested our procedure on data from a past PTM. The LLM was leveraged to extract consistent terminology from PTM questions; the concepts and keywords extracted by the LLM were then matched to the MeSH thesaurus curated by the National Library of Medicine.

Our results indicate that out of 1,639 possible MeSH terms, 1,354 (83%) fully matched descriptors in the MeSH thesaurus, 20 (1%) fully matched entry terms, 52 (3%) partially matched descriptors, 44 (3%) partially matched entry terms, and 169 (10%) did not match any descriptors or entry terms.

In the end, we generated individual feedback on up to 243 medical topics defined by MeSH terms. Furthermore, our approach demonstrated its ability to identify knowledge gaps and problem areas in specific groups. The procedure was most effective with students in the later stages of their studies, as they showed deficiencies in topics covered in earlier semesters.

## Figures and Tables

**Fig. 10:**
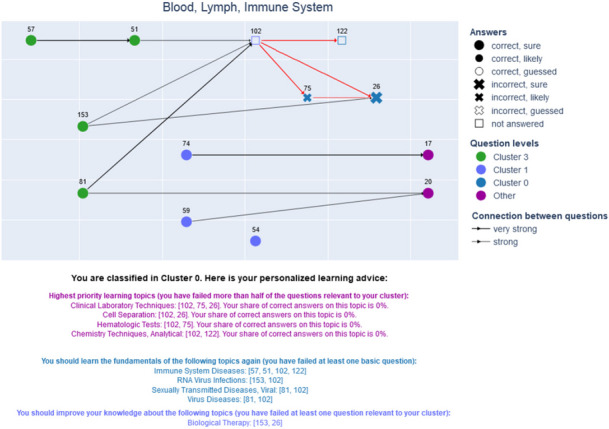
Example of learning guide for the organ system “Blood, Lymph, Immune System”

## Data Availability

The datasets generated during and/or analyzed during the current study are not publicly available for data security reasons but are available from the corresponding author on reasonable request and after approval of the Progress Test cooperation partners and an extended ethical approval.

## Appendix A – Precursor questions

### Definition

We say that question *A* is a precursor question to question *B* if.

A1 $$f(A,B)=P(B=1\vert A=0)/P(B=1\vert A=1)<x$$

where *A* = 1 corresponds to the event of having answered question *A* correctly with confidence level “sure” or “likely”, and *A* = 0 corresponds to any other event excluding response omissions. Only responses by test takers who answered both *A* and *B* were considered.

The ratio $P(B=1\vert A=0)/P(B=1\vert A=1)$ can be understood as the inverse of a relative risk ratio where students for which *A* = 1 were the “exposed group” and the rest were the “unexposed group”. We decided to derive the threshold *x* in (A1) from the effect size categories defined by Sawilowsky [19] on the basis of Cohen’s *d*. We cannot relate the relative risk to Cohen’s *d* via the odds ratio because the odds ratio is symmetrical, while the relative risk is not. The relationship we want to model is non-symmetric, since in general $f(A,B)\ne f(B,A)$*;* moreover, precursor questions do have a hierarchical component that does not fit well with the idea of symmetry. However, we can take the case where $P(B=1\vert A=0)=P(B=0\vert A=1)$ and $P(B=0\vert A=0)=P(B=1\vert A=1)$, for which the precursor question function is symmetric, i.e. $f(A,B)=f(B,A)$ or $P(B=1\vert A=0)/P(B=1\vert A=1)=P(A=1\vert B=0)/P(A=1\vert B=1)$; this allows us to calculate both the odds ratio and Cohen’s *d* for a given value of $f(A,B)$. If $a=P(B=1\vert A=0)=P(B=0\vert A=1)$ and $b=P(B=0\vert A=0)=P(B=1\vert A=1)$, then

A2 $$f\left(A,B\right)=\frac{\frac{a}{\left(a+b\right)}}{\frac{b}{\left(b+a\right)}}=\frac{a}{b}$$

and the odds ratio is:

A3 $$OR=\frac{{b}^{2} }{{a}^{2} }=\frac{1 }{{f(A,B)}^{2} }$$

Since Cohen’s *d* can be obtained from the natural logarithm of an odds ratio by dividing it by 1.81 [20], we can formulate the relationship between the precursor question function and Cohen´s *d* thus:

A4 $$f(A,B)=\sqrt{\frac1{e^{Cohen's\,d\,\cdot\,1.81}}}$$

Applying (A4), the values of $f(A,B)$ associated to medium, large and very large effect sizes as defined by Sawilowsky [19] would be:

- Medium effect size (Cohen’s *d* >0.5): $f(A,B)$<0.636.
- Large effect size (Cohen’s *d* >0.8): $f(A,B)$<0.485.
- Very large effect size (Cohen’s *d* >1.2): $f(A,B)$<0.338.

On account of the characteristics of our data, we have chosen $f(A,B)$ <0.485 as our main threshold for precursor questions. That is, we find it reasonable to state that A is a precursor question of B if $f(A,B)$ <0.485.

### Precursor questions and local independence

The precursor relation function $f(A,B)$ measures the extent to which knowing the answer to question A helps answering question B correctly. It is an effect size function based on the relative risk index; in this sense, one could say that the precursor relation bears some similarities with local dependence as described by Henning [21], since both are derived from conditional probability and are meant to discern whether or not two test questions are empirically related. However, the fact that local dependence is always symmetric, while the precursor relation function is not, highlights the scope of the differences between these two concepts. On one hand, two questions are understood to be locally independent when they are statistically independent as events. On the other hand, precursor relations are born out of the empirical assumption that PTM questions are unlikely to be statistically independent as events; it cannot be guaranteed that each one of them covers a knowledge space that is completely detached to that of other questions. Indeed, the PTM is intended to encompass the entire curriculum of a five-year medical degree course; therefore, it can be expected that there are questions that cover the same topics with varying difficulty levels.

### Similarities and differences with Doignon and Falmagne’s surmise relations

Unlike local dependency measures, the precursor relation function $f(A,B)$ does not gauge the similarity between two questions as a correlation function would do, but rather whether there is an effect-based relationship between these two questions, often of a hierarchical nature. Because of this, one could argue that precursor relations belong to the family of surmise relations posited by Doignon and Falmagne [16]; as a matter of fact, they define surmise relations to mean that “if a student solves question x, it can be surmised that question y can also be solved by that student”. Precursor relations, however, are defined the other way around; they mean that if a student solves question y, the likelihood that this student will also solve question x is higher than otherwise. Moreover, Doignon and Falmagne conceive surmise relationships as binary and transitive, since they find these two characteristics to be useful to construct a knowledge space model based on set theory. The precursor relation is however not transitive; it is binary only if we define $f(A,B)$ to be 1 if *A* and *B* are precursor questions and 0 otherwise.

## Appendix B – Treatment of outputs from large language models

### Evaluating differences between outputs – lemmatization and cosine similarity

The probabilistic layout of LLMs implies that submitting the same input repeatedly does not lead to a unique output; for this reason, we prompted ChatGPT 4.0 to extract MeSH terms in three rounds for every PTM question. To evaluate the differences between the three outputs, we first lemmatized them; that is, we changed them to their non-inflected form, also called “lemma” or “dictionary form”. For example, “write” would be the lemma of the verbal forms “to write”, “writing”, “written” and “wrote”, as well as the nouns “writer” and “writing” and the adjective “written”.

Once lemmatized, we calculated cosine similarity scores between the three outputs. Cosine similarity is a measure of similarity between two non-zero vectors A and B, equivalent to the cosine of the angle between A and B. It is defined as $cos(\theta )=\frac{A\cdot B}{\Vert A\Vert \Vert B\Vert }$ where $A\cdot B$ is the dot product of A and B, and $\Vert A\Vert$ and $\Vert B\Vert$ are the respective lengths of A and B. A value of 1 indicates that the vectors point at the same direction, whereas a value of 0 means they point at orthogonal directions, and a value of -1 means they point at exactly opposite directions.

While the interval of possible values for a cosine is [-1,1], negative values are not used in word counts; therefore, the possible values for cosine similarity in natural language processing go from 0 (implying maximum dissimilarity) to 1 (implying identity). For example, let *q* be a test question for which ChatGPT 4.0 extracts the following keyword sets across three rounds:

- Q1 = {aspirin, salicylates, hydrocarbons, phenol, dyspnea, lungs}
- Q2 = {aspirin, salicylates, hydrocarbon, phenols, dyspnea, respiration}
- Q3 = {aspirin, salicylates, hydrocarbon, lipids, benzene, lung}

After lemmatization, these keyword sets would become:

- Q1 = {aspirin, salicylate, hydrocarbon, phenol, dyspnea, lung}
- Q2 = {aspirin, salicylate, hydrocarbon, phenol, dyspnea, respiration}
- Q3 = {aspirin, salicylate, hydrocarbon, lipid, benzene, lung}

To compute the cosine similarity, we must first determine how often the lemmatized words occur in each set. This is shown in Table 6.

**Table 6:** Frequency of lemmatized words in sets Q1, Q2 and Q3

Set	aspirin	salicylate	hydrocarbon	phenol	dyspnea	lung	respiration	lipid	benzene
Q1	1	1	1	1	1	1	0	0	0
Q2	1	1	1	1	1	0	1	0	0
Q3	1	1	1	0	0	1	0	1	1

We then express the data in the table in the form of numerical vectors:

- Q1 = {1,1,1,1,1,1,0,0,0}
- Q2 = {1,1,1,1,1,0,1,0,0}
- Q3 = {1,1,1,0,0,1,0,1,1}

Finally, we compute the cosine similarities between all possible vector pairs:

$$cos(\mathrm{Q1,Q2})=\frac{5}{6}\cong 0.833$$

$$cos(\mathrm{Q2,Q3})=\frac{3}{6}=0.5$$

$$cos(\mathrm{Q1,Q3})=\frac{3}{6}=0.5$$

### Matching extracted keywords and concepts to terms in the MeSH thesaurus

We matched the keywords and concepts extracted by ChatGPT to the descriptors and entry terms featured in the 2024 MeSH thesaurus, which we obtained from an ASCII file provided for download on https://nlmpubs.nlm.nih.gov/projects/mesh/2024/asciimesh/. Full matches were exact matches between an extracted keyword and a MeSH descriptor or entry term; partial matches between an extracted keyword and a MeSH term were defined as a substring of a keyword that matches a MeSH term, or vice versa. For example, the extracted keyword “Hemoglobin” is a substring of the MeSH descriptor “Hemoglobins”, thus being considered a partial match.

## Appendix C – tf-idf

Tf-idf (acronym of term frequency-inverse document frequency) is a measure of the quantitative importance of terms with respect to documents included in text corpuses, proposed in 1972 by Karen Spärck Jones in her article “A Statistical Interpretation of Term Specificity and Its Application in Retrieval” [15]. It is the product of two separate statistics, term frequency (tf) and inverse document frequency (idf). The term frequency of a term within a document is defined as$tf\left(t,d\right)=\frac{{f}_{t,d}}{{\sum }_{d2}{f}_{t,d2}}$, where the numerator is the number of occurrences of the term in the document, and the denominator is the total number of term occurrences within the document. Thus, the term frequency is equal to the share of term occurrences in the document that correspond to *t*. The inverse document frequency of a term *t* within a text corpus is defined as $idf\left(t,c\right)=log\frac{N}{\left|\{d\right|t\in d,d\in c\}|}$, where the numerator is the total number of documents in the corpus, and the denominator is the number of documents in the corpus that contain the term *t*. Thus, $tf-idf(t,d,c)=\frac{{f}_{t,d}}{{\sum }_{d2}{f}_{t,d2}}log\frac{N}{\left|\{d2\right|t\in d2,d2\in c\}|}$*.*

As an example, we will compare two MeSH terms, “Body temperature changes” and “Skeleton”, which are both related to 10 PTM questions. Their distribution per organ system is however very different, as Table 7 shows.

**Table 7:** Occurrences of the MeSH Terms “Body Temperature Changes”and “Skeleton” per organ system

Organ system	Body Temperature Changes	Skeleton
General Pathology	1	0
Respiratory Organs	1	0
Musculoskeletal System	1	9
Blood, Lymph, Immune System	0	0
Reproductive Organs	0	0
Skin	1	0
Cardiovascular System	2	0
Hormones, Metabolism	0	0
Methodology, Instruments	0	1
Nervous System, Brain, Senses	1	0
Kidney, Urinary Tract	2	0
Mind, Social Issues	1	0
Digestive System	0	0
Cell	0	0

Table 8 shows the tf-idf values obtained for the MeSH terms “Body temperature changes” and “Skeleton”. As expected, 9 of the 10 questions related to the MeSH term “Skeleton” are classified in the organ system “Musculoskeletal system”. Questions related to the MeSH term “Body Temperature Changes” are however much more evenly distributed; there is no organ system that contains more than two questions connected to this MeSH term. The maximum number of questions associated with a single organ system is 9 for “Skeleton” and 2 “Body Temperature Changes”, which implies a ratio of 4.5 to 1. After computing tf-idf values, we see that the maximum value for “Skeleton” is 0.0448, while such value for “Body Temperature changes” amounts to 0.0028, which corresponds to a ratio of 16 to 1. Therefore, in this example tf-idf values highlight the attachment of MeSH terms to organ systems rather more strongly than a simple occurrence count.

**Table 8:** Tf-idf values for the MeSH Terms”Body Temperature Changes” and “Skeleton” per organ system

Organ system	Body Temperature Changes	Skeleton
General Pathology	0.0016	0
Respiratory Organs	0.0008	0
Musculoskeletal System	0.0014	0.0448
Blood, Lymph, Immune System	0	0
Reproductive Organs	0	0
Skin	0.0026	0
Cardiovascular System	0.0014	0
Hormones, Metabolism	0	0
Methodology, Instruments	0	0.0102
Nervous System, Brain, Senses	0.0013	0
Kidney, Urinary Tract	0.0028	0
Mind, Social Issues	0.0008	0
Digestive System	0	0
Cell	0	0

## Appendix D – Example of visual feedback

We provide here a prototypical example of visual feedback generated with Plotly 5.13.0 following the guidelines outlined in this study. In this example, a PTM participant shows knowledge gaps in some areas of the organ system “Blood, Lymph, Immune System”, particularly those related to laboratory techniques. The suggested learning topics are listed below the diagram; the red arrows in the diagram indicate which questions are related to the highest priority learning topics.
